# Supplementary material for: Dbf4-dependent kinase promotes cell cycle controlled resection of DNA double-strand breaks and repair by homologous recombination
Source: Nat Commun. 2024 Apr 3;15:2890. doi: 10.1038/s41467-024-46951-z (PMC10991553; doi:10.1038/s41467-024-46951-z)
Supplement: Supplementary file 1 — Supplementary Information [file 41467_2024_46951_MOESM1_ESM.pdf]

Supplementary Information

for

Dbf4-dependent kinase promotes cell-cycle controlled resection of DNA double-strand breaks and repair by homologous recombination

Lorenzo Galanti, Martina Peritore, Robert Gnügge, Elda Cannavo, Johannes Heipke, Maria Dilia Palumbieri, Barbara Steigenberger, Lorraine S. Symington, Petr Cejka, Boris Pfander

Supplementary Figures 1-5 and Supplementary Tables 1-3

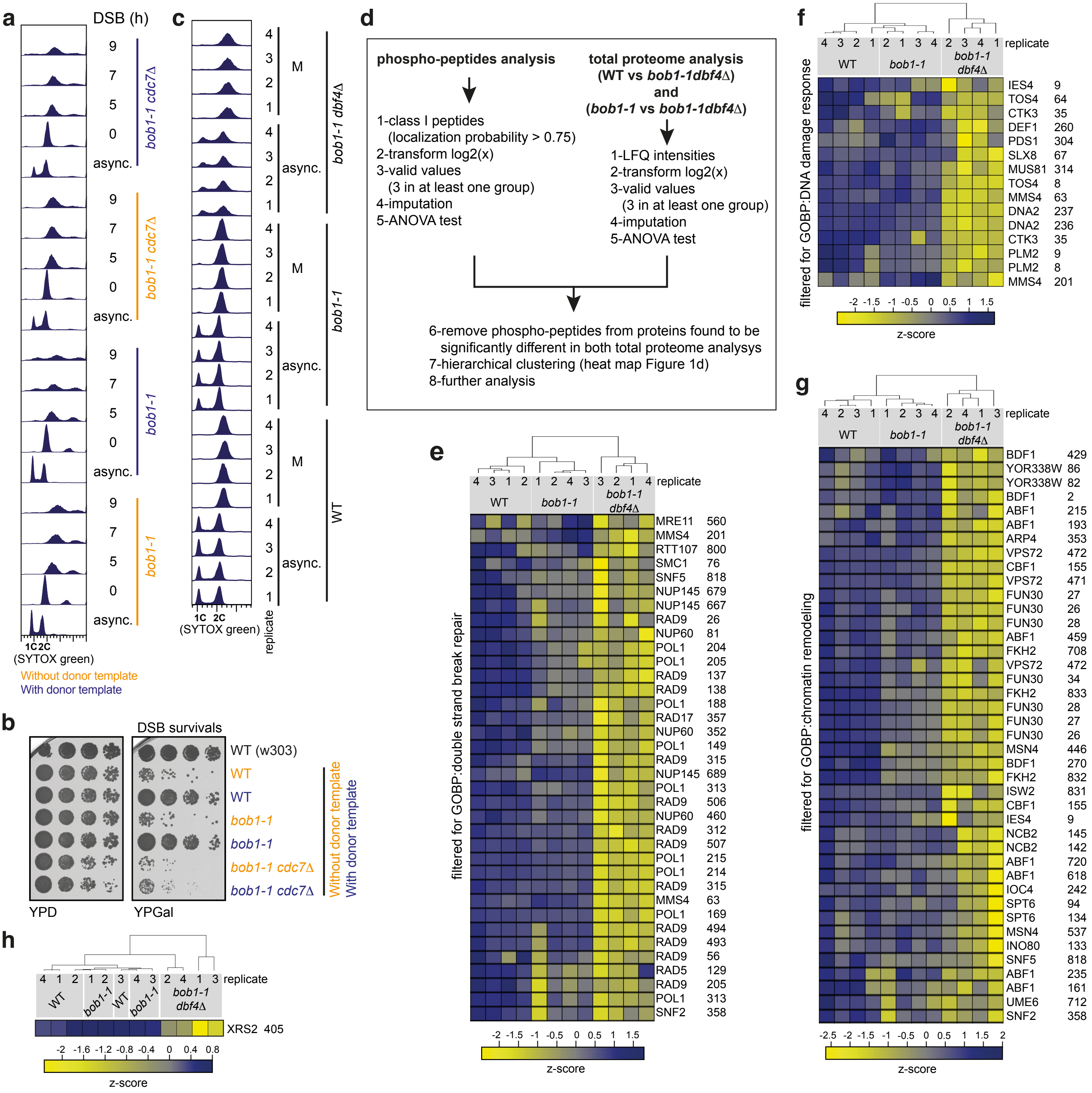
a
DSB (h)
9
7
5
0
async.
bob1-1 cdc7Δ
bob1-1
1C 2C
(SYTOX green)
Without donor template
With donor template
c
4
3
2
1
M
async.
bob1-1 dbf4Δ
bob1-1
WT
replicate
d
phospho-peptides analysis
1-class I peptides
(localization probability > 0.75)
2-transform log2(x)
3-valid values
(3 in at least one group)
4-imputation
5-ANOVA test
total proteome analysis
(WT vs bob1-1dbf4Δ)
and
(bob1-1 vs bob1-1dbf4Δ)
1-LFQ intensities
2-transform log2(x)
3-valid values
(3 in at least one group)
4-imputation
5-ANOVA test
6-remove phospho-peptides from proteins found to be significantly different in both total proteome analysys
7-hierarchical clustering (heat map Figure 1d)
8-further analysis
b
DSB survivals
WT (w303)
WT
WT
bob1-1
bob1-1
bob1-1 cdc7Δ
bob1-1 cdc7Δ
Without donor template
With donor template
YPD
YPGal
e
4 3 1 2 1 2 4 3 3 2 1 4 replicate
WT
bob1-1
bob1-1 dbf4Δ
filtered for GOBP:double strand break repair
MRE11 560
MMS4 201
RTT107 800
SMC1 76
SNF5 818
NUP145 679
NUP145 667
RAD9 26
NUP60 81
POL1 204
POL1 205
RAD9 137
RAD9 138
POL1 188
RAD17 357
NUP60 352
POL1 149
RAD9 315
NUP145 689
POL1 313
RAD9 506
NUP60 460
RAD9 312
RAD9 507
POL1 215
POL1 214
RAD9 315
MMS4 63
POL1 169
RAD9 494
RAD9 493
RAD9 56
RAD5 129
RAD9 205
POL1 313
SNF2 358
-2 -1.5 -1 -0.5 0 0.5 1 1.5
z-score
f
4 3 2 1 2 1 3 4 2 3 4 1 replicate
WT
bob1-1
bob1-1 dbf4Δ
filtered for GOBP:DNA damage response
IES4 9
TOS4 64
CTK3 35
DEF1 260
PDS1 304
SLX8 67
MUS81 314
TOS4 8
MMS4 63
DNA2 237
DNA2 236
CTK3 35
PLM2 9
PLM2 8
MMS4 201
-2 -1.5 -1 -0.5 0 0.5 1 1.5
z-score
g
4 2 3 1 1 2 3 4 2 4 1 3 replicate
WT
bob1-1
bob1-1 dbf4Δ
filtered for GOBP:chromatin remodeling
BDF1 429
YOR338W 86
YOR338W 82
BDF1 2
ABF1 215
ABF1 193
ARP4 353
VPS72 472
CBF1 155
VPS72 471
FUN30 27
FUN30 26
FUN30 28
ABF1 459
FKH2 708
VPS72 472
FUN30 34
FKH2 833
FUN30 28
FUN30 27
FUN30 26
MSN4 446
BDF1 270
FKH2 832
ISW2 831
CBF1 155
IES4 9
NCB2 145
NCB2 142
ABF1 720
ABF1 618
IOC4 242
SPT6 94
SPT6 134
MSN4 537
INO80 133
SNF5 818
ABF1 235
ABF1 161
UME6 712
SNF2 358
-2.5 -2 -1.5 -1 -0.5 0 0.5 1 1.5 2
z-score
h
4 1 2 1 2 3 4 3 2 4 1 3 replicate
WT
bob1-1
WT
bob1-1
bob1-1 dbf4Δ
XRS2 405
-2 -1.6 -1.2 -0.8 -0.4 0 0.4 0.8
z-score

Supplementary Figure 1 – Related to Figure 1

(a) DNA content measurement by flow cytometry to confirm M-phase cell cycle arrest. Data are representative of n=3 biological replicates. Related to experiment shown in Fig. 1c. ‘async.’ stands for asynchronous.
(b) DDK mutants are defective in survival by HR. Using system from Fig. 1b (to monitor HR-dependent cell survival), five-fold serial dilutions of the reported strains (with or without the donor template) were spotted on YPD (as control) or YPGal (for chronic pGAL-HO-induction) plates. Data are representative of n=3 biological replicates.
(c) DNA content measurement by flow cytometry to confirm M-phase cell cycle arrest. Related to experiment shown in Fig. 1d. ‘async.’ stands for asynchronous.
(d) Pipeline for the analysis of the phospho-proteome (and total proteome) using Perseus (v1.6.5.0).
(e-g) Heat maps depicting the z-score, highlighting phospho-peptides from the DDK cluster after filtering in Perseus (v1.6.5.0) for (e) GOBP: double-strand break repair (GO: 0006302), (f) GOBP: DNA damage response (GO:0006974) and (g) GOBP: chromatin remodeling (GO:0006338). Gene name and modified residues are reported. GOBP = Gene Ontology Biological Process. Related to experiment shown in Fig. 1d.
(h) Heat map depicting the z-score, highlighting the Xrs2 phospho-peptide identified in the DDK cluster. Related to experiment shown in Fig. 1d.
Source data are provided as a Source Data file.

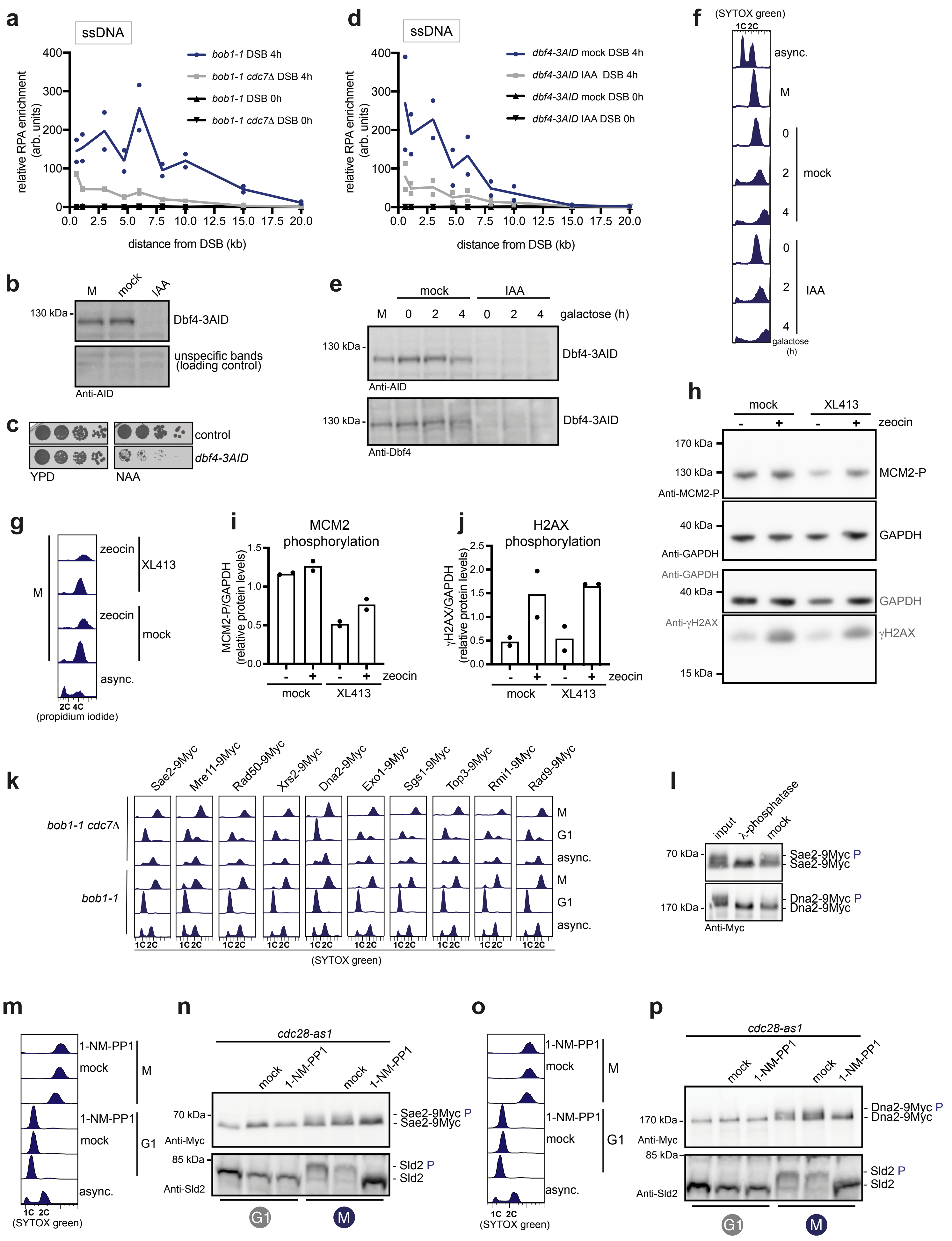
a
ssDNA
relative RPA enrichment (arb. units)
bob1-1 DSB 4h
bob1-1 cdc7Δ DSB 4h
bob1-1 DSB 0h
bob1-1 cdc7Δ DSB 0h
distance from DSB (kb)
b
M
mock
IAA
130 kDa
Dbf4-3AID
unspecific bands (loading control)
Anti-AID
c
control
dbf4-3AID
YPD
NAA
d
ssDNA
relative RPA enrichment (arb. units)
dbf4-3AID mock DSB 4h
dbf4-3AID IAA DSB 4h
dbf4-3AID mock DSB 0h
dbf4-3AID IAA DSB 0h
distance from DSB (kb)
e
mock
IAA
galactose (h)
130 kDa
Dbf4-3AID
Anti-AID
Anti-Dbf4
f
(SYTOX green)
1C 2C
async.
M
mock
IAA
galactose (h)
g
zeocin
XL413
mock
M
async.
2C 4C
(propidium iodide)
i
MCM2 phosphorylation
MCM2-P/GAPDH (relative protein levels)
zeocin
mock
XL413
j
H2AX phosphorylation
γH2AX/GAPDH (relative protein levels)
zeocin
mock
XL413
h
mock
XL413
zeocin
170 kDa
130 kDa
Anti-MCM2-P
MCM2-P
40 kDa
Anti-GAPDH
GAPDH
Anti-γH2AX
γH2AX
15 kDa
k
Sae2-9Myc
Mre11-9Myc
Rad50-9Myc
Xrs2-9Myc
Dna2-9Myc
Exo1-9Myc
Sgs1-9Myc
Top3-9Myc
Rmi1-9Myc
Rad9-9Myc
bob1-1 cdc7Δ
bob1-1
M
G1
async.
1C 2C
(SYTOX green)
l
input
λ-phosphatase
mock
70 kDa
Sae2-9Myc P
Sae2-9Myc
170 kDa
Dna2-9Myc P
Dna2-9Myc
Anti-Myc
m
1-NM-PP1
mock
M
G1
async.
1C 2C
(SYTOX green)
n
cdc28-as1
mock
1-NM-PP1
70 kDa
Sae2-9Myc P
Sae2-9Myc
Anti-Myc
85 kDa
Sld2 P
Sld2
Anti-Sld2
G1
M
o
1-NM-PP1
mock
M
G1
async.
1C 2C
(SYTOX green)
p
cdc28-as1
mock
1-NM-PP1
170 kDa
Dna2-9Myc P
Dna2-9Myc
Anti-Myc
85 kDa
Sld2 P
Sld2
Anti-Sld2
G1
M

Supplementary Figure 2 – Related to Figure 2

(a) Enrichment of RPA in *bob1-1* and *bob1-1cdc7Δ* cells, monitored via ChIP-qPCR at an HO-induced DSB at the *MAT* locus using several primer pairs (up to 20 kb downstream the DSB). Experiments were performed using cells arrested in M-phase. n=2, values of biological replicates are shown; curves display the mean values.
(b-c) Controls to test the Dbf4-3AID system. (b) M-phase arrested *dbf4-3AID* cells were either mock treated or treated with 1 mM IAA. (c) Five-fold serial dilutions of WT or *dbf4-3AID* cells were spotted on YPD plates (as control) or YPD plates supplemented with 1 mM NAA.
(d) Same as in (a), but using *dbf4-3AID* cells mock or treated with 1 mM IAA prior DSB induction.
(e-f) (e) Western blot with anti-AID and an anti-Dbf4 antibodies to control Dbf4-3AID degradation in IAA-treated cells. (f) DNA content measurement by flow cytometry to confirm M-phase arrest. 'async.' stands for asynchronous. Data are representative of n=4 biological replicates. Related to experiment shown in Fig. 2d-e.
(g) DNA content measurement by flow cytometry to confirm M-phase cell cycle arrest. Data are representative of n=2 biological replicates. Related to experiment shown in Fig. 2f. 'async.' stands for asynchronous.
(h) Western blot of samples to monitor DDK-inhibition using MCM2-phosphoS53 (MCM2-P) as a proxy for DDK activity and γH2AX as a proxy for DNA damage induction. Data are representative of n=2 biological replicates. Related to experiment shown in Fig. 2f.
(i-j) (i) Quantification of the MCM2-phosphoS53 (MCM2-P) signal relative to GAPDH as loading control. (j) Quantification of the γH2AX signal relative to GAPDH as loading control. n=2, box plot shows mean with values of biological replicates. Related to experiment shown in Fig. 2f.
(k) DNA content measurement by flow cytometry to confirm G1 or M-phase cell cycle arrest of the reported strains. Data are representative of n=2-4 biological replicates. Related to experiment shown in Fig. 2g.
(l) Sae2 and Dna2 gel-shift is sensitive to λ-phosphatase. Soluble extracts from M-phase cells expressing Sae2-9Myc or Dna2-9Myc (input) were split to be mock or λ-phosphatase-treated and the gel-shift monitored on gel. Data are representative of n=2 technical replicates.
(m-p) *cdc28-as1* cells carrying Sae2-9Myc (m-n) or Dna2-9Myc (o-p) were arrested in G1 or M-phase and either mock treated or treated with 1.5 μM 1-NM-PP1 to inhibit CDK. Upon CDK inhibition, (n) the Sae2 phospho-shift is reduced, while (p) the Dna2 phospho-shift is largely abolished. Sld2-CDK-dependent phospho-shift was used as a control for CDK inhibition. DNA content measurement by flow cytometry confirmed G1 or M-phase cell cycle arrest of cells expressing Sae2-9Myc (m) or Dna2-9Myc (o). Data are representative of n=3 biological replicates. 'async.' stands for asynchronous.
Source data are provided as a Source Data file.

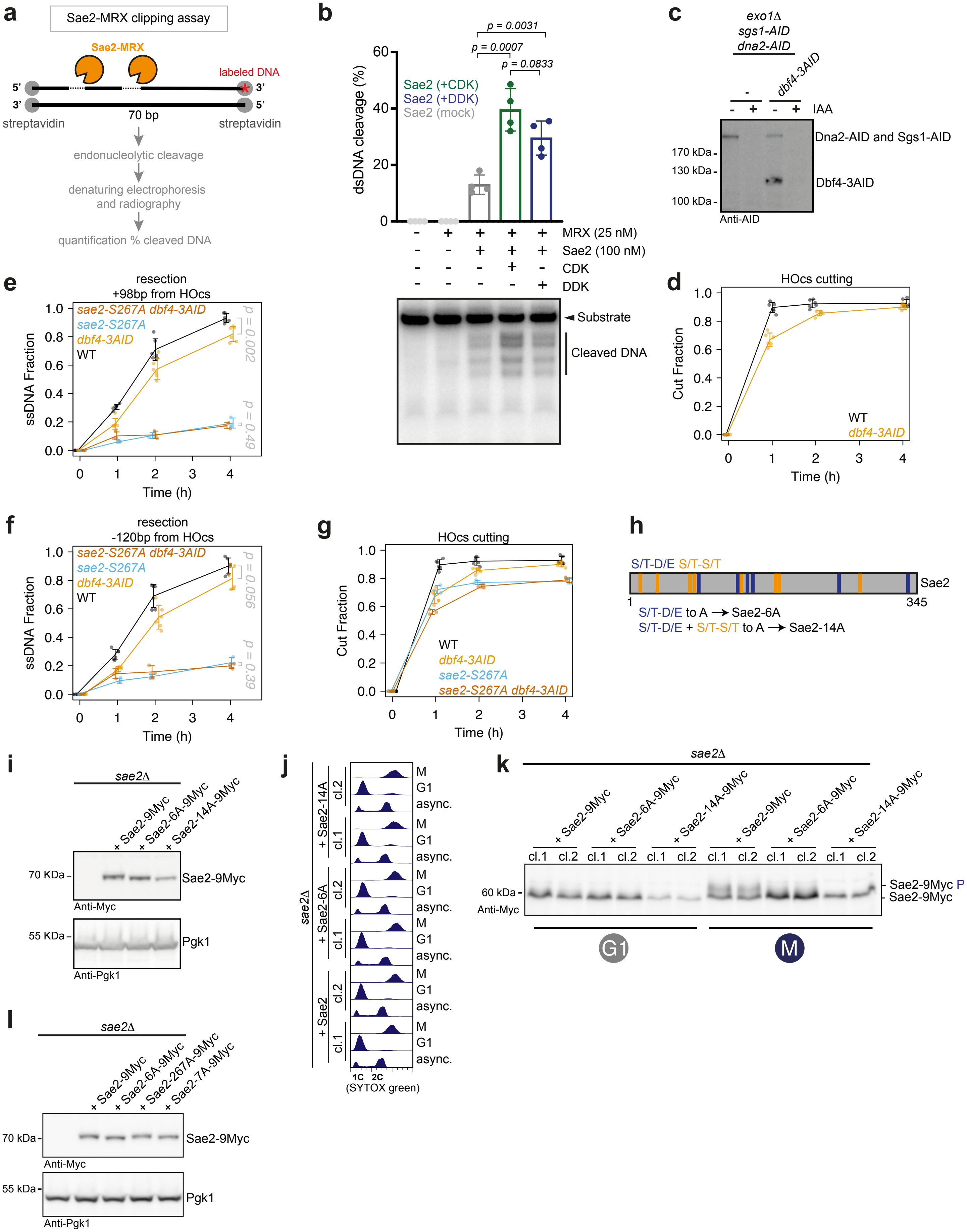
a
Sae2-MRX clipping assay
Sae2-MRX
labeled DNA
5'
3'
3'
5'
70 bp
streptavidin
streptavidin
endonucleolytic cleavage
denaturing electrophoresis and radiography
quantification % cleaved DNA
b
p = 0.0031
p = 0.0007
p = 0.0833
dsDNA cleavage (%)
Sae2 (+CDK)
Sae2 (+DDK)
Sae2 (mock)
MRX (25 nM)
Sae2 (100 nM)
CDK
DDK
Substrate
Cleaved DNA
c
exo1Δ
sgs1-AID
dna2-AID
dbf4-3AID
IAA
170 kDa
130 kDa
100 kDa
Dna2-AID and Sgs1-AID
Dbf4-3AID
Anti-AID
d
HOcs cutting
Cut Fraction
WT
dbf4-3AID
Time (h)
e
resection
+98bp from HOcs
sae2-S267A dbf4-3AID
sae2-S267A
dbf4-3AID
WT
p = 0.002
p = 0.49
ssDNA Fraction
Time (h)
f
resection
-120bp from HOcs
sae2-S267A dbf4-3AID
sae2-S267A
dbf4-3AID
WT
p = 0.056
p = 0.39
ssDNA Fraction
Time (h)
g
HOcs cutting
Cut Fraction
WT
dbf4-3AID
sae2-S267A
sae2-S267A dbf4-3AID
Time (h)
h
S/T-D/E S/T-S/T
Sae2
1
345
S/T-D/E to A → Sae2-6A
S/T-D/E + S/T-S/T to A → Sae2-14A
i
sae2Δ
+ Sae2-9Myc
+ Sae2-6A-9Myc
+ Sae2-14A-9Myc
70 KDa
Sae2-9Myc
Anti-Myc
55 KDa
Pgk1
Anti-Pgk1
j
sae2Δ
+ Sae2-14A
+ Sae2-6A
+ Sae2
cl.1
cl.2
M
G1
async.
1C
2C
(SYTOX green)
k
sae2Δ
+ Sae2-9Myc
+ Sae2-6A-9Myc
+ Sae2-14A-9Myc
cl.1
cl.2
60 kDa
Anti-Myc
Sae2-9Myc P
Sae2-9Myc
G1
M
l
sae2Δ
+ Sae2-9Myc
+ Sae2-6A-9Myc
+ Sae2-267A-9Myc
+ Sae2-7A-9Myc
70 kDa
Sae2-9Myc
Anti-Myc
55 kDa
Pgk1
Anti-Pgk1

Supplementary Figure 3 – Related to Figure 3

(a) Scheme of the clipping assay used to monitor endonucleolytic clipping by Sae2-MRX. ^{32}P-labelled linear DNA with streptavidin blocked ends was used as a substrate of Sae2-MRX. Endonucleolytic DNA cleavage was monitored via the appearance of cleaved DNA products after denaturing electrophoresis. Sae2 was purified separately from MRX, allowing to pre-phosphorylate (or mock-treat) Sae2 before reconstituting the Sae2-MRX complex and monitor its endonucleolytic activity.
(b) Sae2-MRX endonucleolytic activity is slightly higher after CDK phosphorylation of Sae2 compared to DDK phosphorylation. Sae2 was phosphorylated by CDK, DDK or mock treated and added to the MRX complex. Sae2-MRX mediated endonucleolytic clipping of DNA was then monitored on denaturing gels. Top, quantification of the DNA cleavage products; bottom, a representative experiment, n=4, box plot depicts mean with values of replicates, error bars denote SD. Reported *p* values were calculated using a two-tailed unpaired t-test.
(c) Western blot to confirm Dbf4-3AID degradation after treatment with IAA in the strains used for the experiment shown in Fig. 3g-h. Cells were arrested in M-phase and treated with 1 mM IAA. Samples were then loaded on a gel to monitor Dbf4-3AID. Note that Dna2-AID and Sgs1-AID run at the same height and are both degraded after IAA treatment as well.
(d) Cutting efficiency of the HO nuclease at the *MAT* locus measured via qPCR. The cut efficiency is used to normalize the amount of ssDNA to the amount of cut DNA. n=6 biological replicates, shown is mean with values of replicates, error bars denote SD. Related to the experiment shown in Fig. 3g-h.
(e-g) Sae2-MRX mediated short-range resection is reduced upon depletion of Dbf4 and largely abolished in *sae2-S267A* mutants. ssDNA accumulation upon DSB induction measured at sites 98 bp downstream (e) and 120 bp upstream (f) the DSB via qPCR after digestion with restriction nucleases RsaI and MseI, respectively. (g) Cutting efficiency of the HO nuclease at the *MAT* locus measured via qPCR. The cut efficiency is used to normalize the amount of ssDNA to the amount of cut DNA. n=6 (WT and *dbf4-3AID*), n=3 (*sae2-S267A* and *sae2-S267A dbf4-3AID*) biological replicates. Shown is mean with values of replicates, error bars denote SD. Reported *p* values were calculated using a two-tailed unpaired t-test. The data for WT and *dbf4-3AID* in Supplementary Fig. 3e, Supplementary Fig. 3f and Supplementary Fig. 3g are the same as in Fig. 3g, Fig. 3h and Supplementary Fig. 3d, respectively.
(h) Scheme of Sae2 highlighting S/T-D/E and S/T-S/T sites.
(i) Western blot to monitor total protein levels of Sae2, Sae2-6A and Sae2-14A from *sae2Δ* strains complemented with ectopically expressed Sae2 variants 9Myc tagged. Pgk1 is used as a loading control. Sae2-9Myc and the 6A mutant appear to have similar levels, while the 14A mutant appear to have slightly reduced total protein levels.
(j) DNA content measurement by flow cytometry confirmed G1 or M-phase cell cycle arrest of cells expressing Sae2-9Myc variants. Related to experiment shown in Supplementary Fig. 3k. 'async.' stands for asynchronous.

(k) Phospho-shift of Sae2-6A and Sae2-14A mutants. Cells of indicated strains were arrested in G1 or M-phase to monitor the Sae2 phospho-shift. Data are representative of n=3 biological replicates. See also Supplementary Fig. 3j.
(l) Western blot to monitor total protein levels of Sae2, Sae2-6A, Sae2-S267A and Sae2-7A (S267A + 6A) from *sae2*Δ strains complemented with ectopically expressed Sae2 variants 9Myc tagged. Pgk1 is used as a loading control. Source data are provided as a Source Data file.

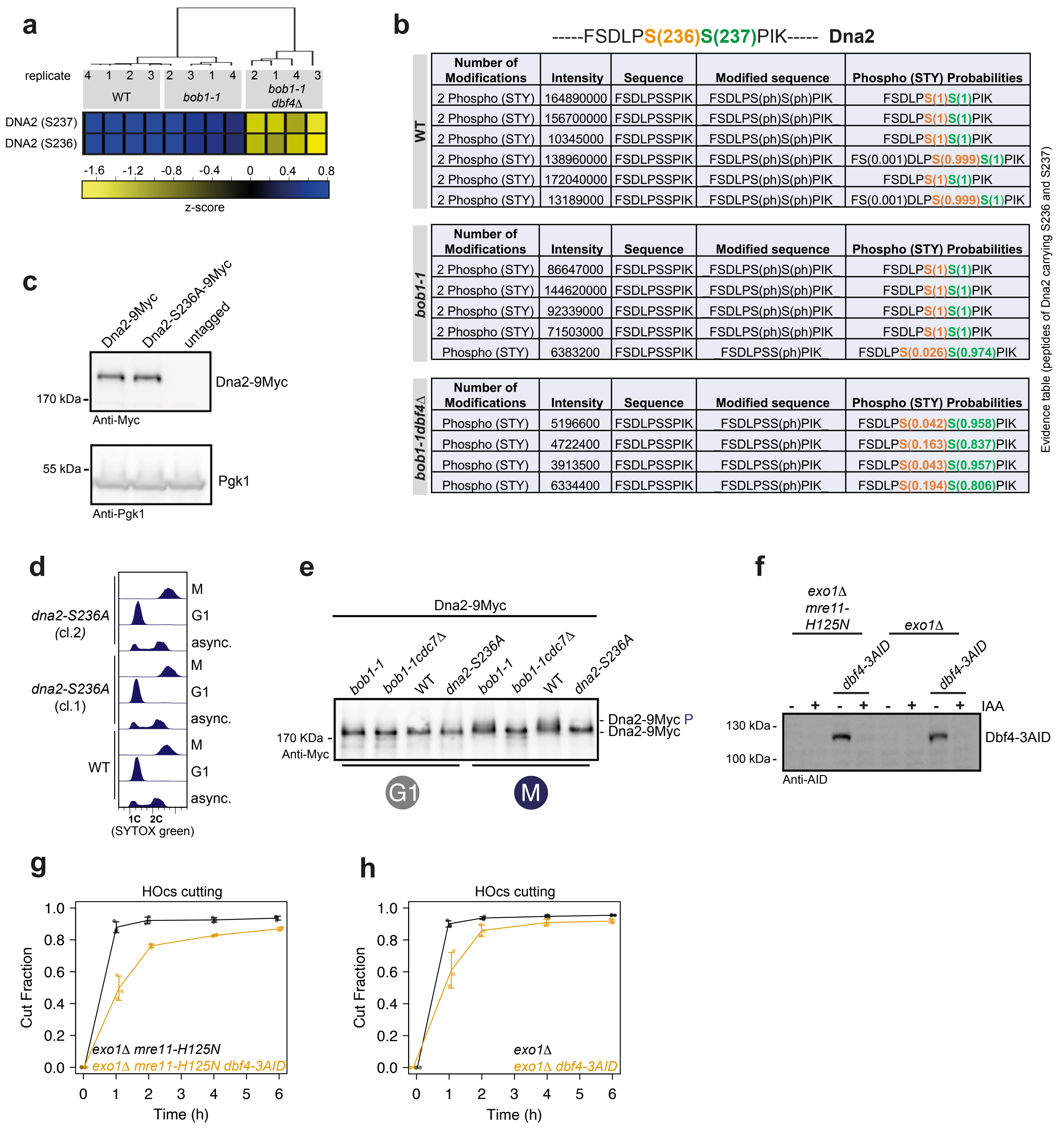

WT

Number of Modifications	Intensity	Sequence	Modified sequence	Phospho (STY) Probabilities
2 Phospho (STY)	164890000	FSDLPSSPIK	_FSDLPS(ph)S(ph)PIK_	FSDLPS(1)S(1)PIK
2 Phospho (STY)	156700000	FSDLPSSPIK	_FSDLPS(ph)S(ph)PIK_	FSDLPS(1)S(1)PIK
2 Phospho (STY)	10345000	FSDLPSSPIK	_FSDLPS(ph)S(ph)PIK_	FSDLPS(1)S(1)PIK
2 Phospho (STY)	138960000	FSDLPSSPIK	_FSDLPS(ph)S(ph)PIK_	FS(0.001)DLPS(0.999)S(1)PIK
2 Phospho (STY)	172040000	FSDLPSSPIK	_FSDLPS(ph)S(ph)PIK_	FSDLPS(1)S(1)PIK
2 Phospho (STY)	13189000	FSDLPSSPIK	_FSDLPS(ph)S(ph)PIK_	FS(0.001)DLPS(0.999)S(1)PIK

bob1-1

Number of Modifications	Intensity	Sequence	Modified sequence	Phospho (STY) Probabilities
2 Phospho (STY)	86647000	FSDLPSSPIK	_FSDLPS(ph)S(ph)PIK_	FSDLPS(1)S(1)PIK
2 Phospho (STY)	144620000	FSDLPSSPIK	_FSDLPS(ph)S(ph)PIK_	FSDLPS(1)S(1)PIK
2 Phospho (STY)	92339000	FSDLPSSPIK	_FSDLPS(ph)S(ph)PIK_	FSDLPS(1)S(1)PIK
2 Phospho (STY)	71503000	FSDLPSSPIK	_FSDLPS(ph)S(ph)PIK_	FSDLPS(1)S(1)PIK
Phospho (STY)	6383200	FSDLPSSPIK	_FSDLPSS(ph)PIK_	FSDLPS(0.026)S(0.974)PIK

bob1-1dbf4Δ

Number of Modifications	Intensity	Sequence	Modified sequence	Phospho (STY) Probabilities
Phospho (STY)	5196600	FSDLPSSPIK	_FSDLPSS(ph)PIK_	FSDLPS(0.042)S(0.958)PIK
Phospho (STY)	4722400	FSDLPSSPIK	_FSDLPSS(ph)PIK_	FSDLPS(0.163)S(0.837)PIK
Phospho (STY)	3913500	FSDLPSSPIK	_FSDLPSS(ph)PIK_	FSDLPS(0.043)S(0.957)PIK
Phospho (STY)	6334400	FSDLPSSPIK	_FSDLPSS(ph)PIK_	FSDLPS(0.194)S(0.806)PIK

Supplementary Figure 4 – Related to Figure 4

(a) Highlight of serine 236 and serine 237 of Dna2. Data from phospho-proteomic experiment of Fig. 1d. Heat-map depicts the z-score.
(b) Evidence for identified phospho-peptides carrying serine 236 and serine 237 of Dna2. Serine 236 is highlighted in orange while serine 237 is highlighted in green. Double-phosphorylated peptides (S236-phospho/S237-phospho) are identified in all replicates of WT and *bob1-1* controls (with probability of 100% for both serine). In contrast, only singly phosphorylated peptides are identified in all replicates of the *bob1-1dbf4*Δ strain, with the phosphorylation always assigned with high probability (80% up to 95%) to serine 237, suggesting that DDK is required specifically for the phosphorylation of serine 236.
(c) Western blot to monitor total protein levels of Dna2-9Myc and Dna2-S236A-9Myc. Pgk1 is used as a loading control. Dna2-9Myc and Dna2-S236A-9Myc have similar total protein levels.
(d)) DNA content measurement by flow cytometry confirmed G1 or M-phase cell cycle arrest of cells expressing Dna2-9Myc variants. Related to experiment shown in Fig. 4c. 'async.' stands for asynchronous.
(e) Serine 236 mutation to alanine affects the Dna2 phosphorylation shift to an extent similar to the lack of DDK. Cells of the indicated strains were arrested in G1 or M-phase and samples collected and loaded on a gel to monitor the Dna2 phospho-shift. Data are representative of n=2 biological replicates.
(f) Western blot to confirm Dbf4-3AID degradation after treatment with IAA in the strains used for experiments shown in Fig. 4e-f. Cells were arrested in M-phase and treated with 1 mM IAA. Samples were then loaded on gel to monitor Dbf4-3AID.
(g-h) Cutting efficiency of the HO nuclease at the *MAT* locus measured via qPCR. The cut efficiency is used to normalize the amount of ssDNA to the amount of cut DNA. n=3 biological replicates, shown is mean with values of replicates, error bars denote SD. (g) Related to the experiment shown in Fig. 4e. (h) Related to the experiment shown in Fig. 4f.
Source data are provided as a Source Data file.

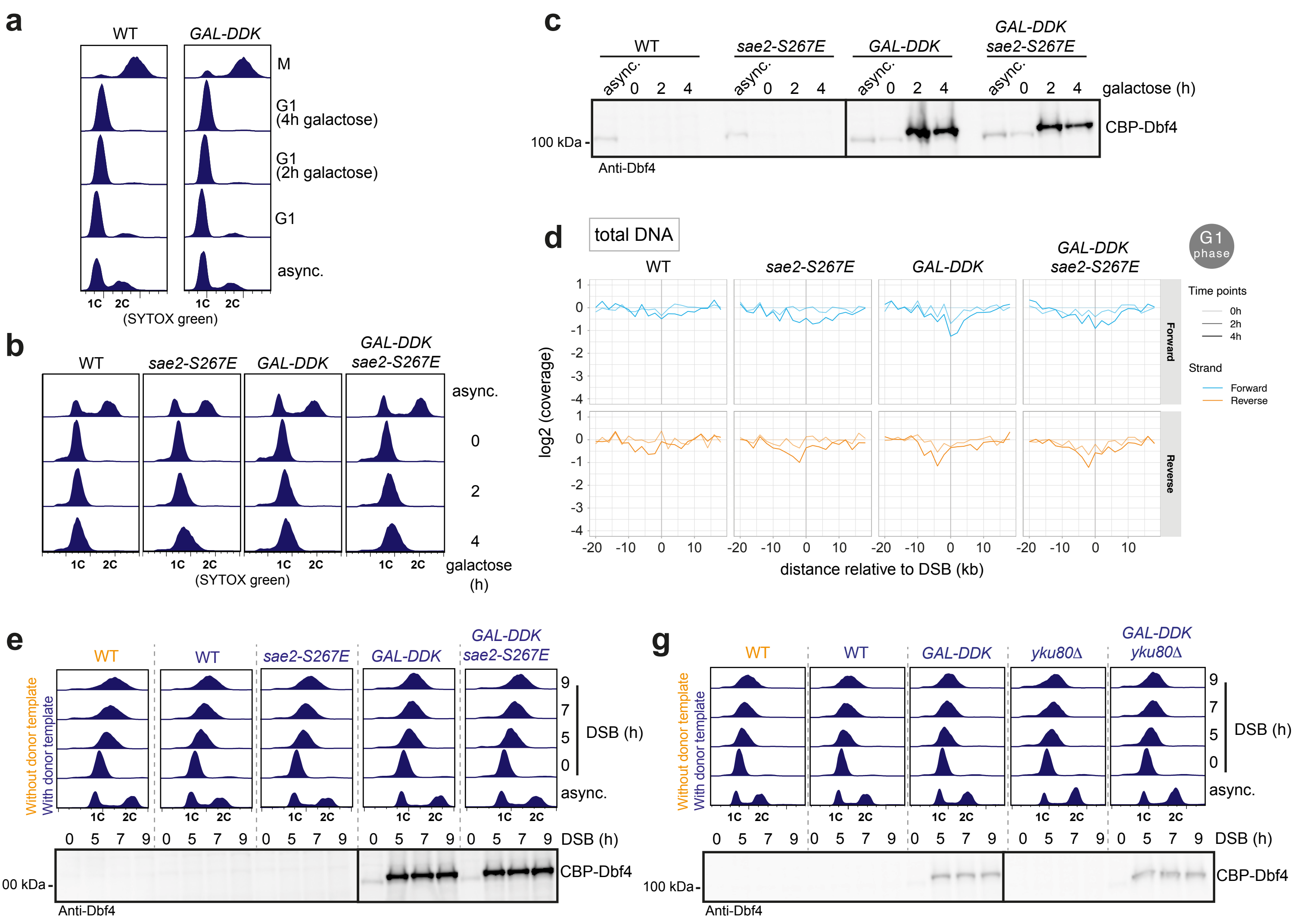

f

% G1 cells	Exp.1	Exp.2	Exp.3
WT	89.9	96.4	95.1
sae2-S267E	94.7	95.2	90.8
GAL-DDK	91.5	93.7	93.1
GAL-DDK sae2-S267E	91.8	94.6	95.7

h

% G1 cells	Exp.1	Exp.2	Exp.3
WT	94.0	95.7	92.7
GAL-DDK	91.5	93.1	94.8
yku80Δ	90.6	92.4	92.6
GAL-DDK yku80Δ	92.6	90.2	87.2

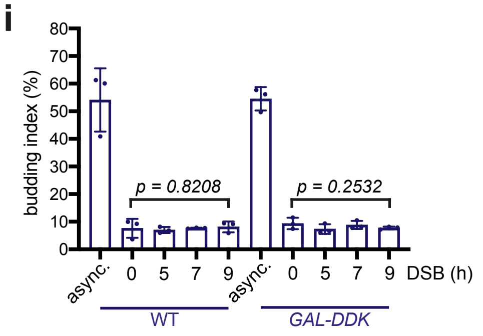

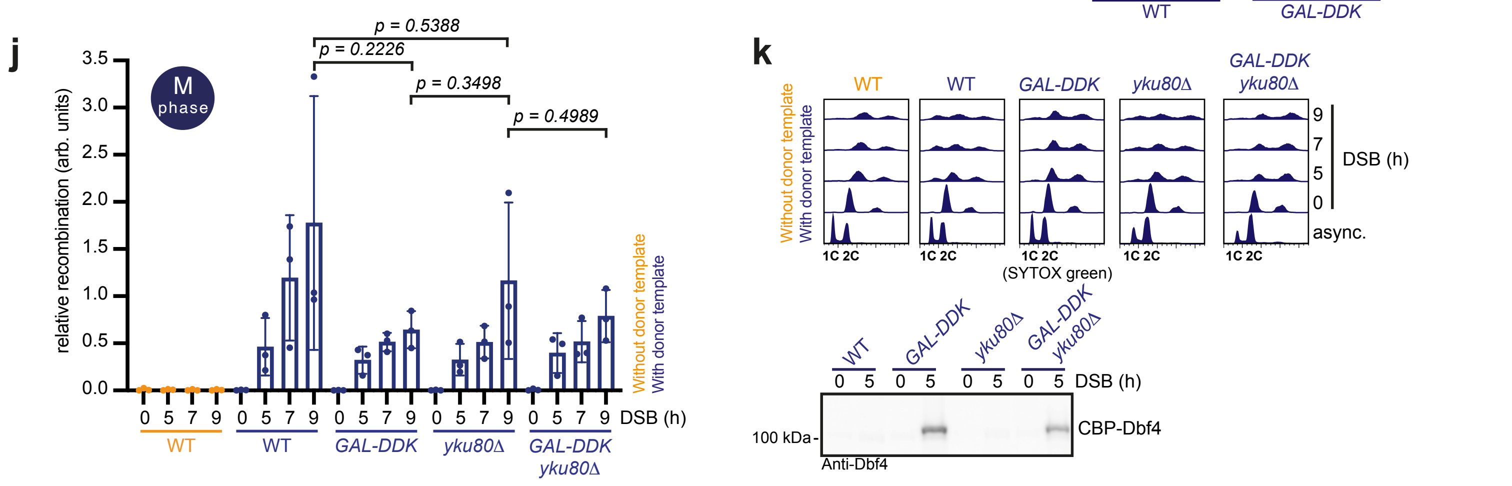

Supplementary Figure 5 – Related to Figure 5

(a) DNA content measurement by flow cytometry to confirm G1 or M-phase cell cycle arrest of the respective strains. Data are representative of n=2 biological replicates. Related to experiment shown in Fig. 5b. ‘async.’ stands for asynchronous.
(b-c) (b) DNA content measurement by flow cytometry to confirm G1 cell cycle arrest of the respective strains. ‘async.’ stands for asynchronous. (c) Western blot to confirm DDK expression (Dbf4 used as representative subunit). Data are representative of n=2 biological replicates. Related to experiment shown in Fig. 5c, Supplementary Fig. 5d.
(d) Total DNA shows limited loss of 5’ DNA strands, consistent with end resection being inhibited in G1. A moderate increase in the loss of 5’ DNA strands is observed in G1 cells expressing DDK (or *sae2-S267E* or the *sae2-S267E GAL-DDK* combination). Strand-specific read coverage is normalized to read coverage before DSB induction. Data are representative of n=2 biological replicates. Related to experiment shown in Fig. 5c.
(e and g) Top, DNA content measurement by flow cytometry to confirm G1-phase cell cycle arrest of the respective strains. Bottom, western blot to confirm expression of DDK in G1 (Dbf4 used as representative subunit). Data are representative of n=3 biological replicates. (e) Related to experiment shown in Fig. 5d. (g) Related to experiment shown in Fig. 5e. ‘async.’ stands for asynchronous.
(f and h) The G1 peak measured by flow cytometry (from 0 hours DSB induction samples) was quantified using FLowJO (V.10.5.3) to retrieve the percentage of G1 cells at the point of DBS induction for the reported strains (in the different biological replicates). Note that cells were kept arrested in G1 throughout the experiment. (f) Related to experiments shown in Fig. 5d. (h) Related to experiment shown in Fig. 5e.
(i) Budding index percentage of WT and *GAL-DDK* cells from experiments shown if Fig. 5d. See also Supplementary Fig. 5e-f. n=3, box plot shows mean with values of biological replicates, error bars denote SD. Reported *p* values were calculated using a two-tailed unpaired t-test.
(j) qPCR analysis of HR upon DSB using system as in Fig. 1b in cells arrested in M-phase. WT cells lacking the donor template are used as negative control n=3, box plot shows mean with values of biological replicates, error bars denote SD. Reported *p* values were calculated using a two-tailed unpaired t-test.
(k) Top, DNA content measurement by flow cytometry to confirm M-phase cell cycle arrest of the respective strains. ‘async.’ stands for asynchronous. Bottom, western blot to confirm expression of DDK (Dbf4 used as representative subunit). Data are representative of n=3 biological replicates. Related to experiment shown in Supplementary Fig. 5j.
Source data are provided as a Source Data file.

Supplementary Table 1 – Plasmids used in this study

Plasmid name	Vector	Insert	Source/Reference
pLG23	pRS306	pGAL1-10 CBP-DBF4/CDC7	this study
p1652	pRS304	pGAL1-10 CBP-DBF4/CDC7	On et al., 2014 (Ref. 88)
pLG25	pRS306	pSAE2-SAE2-9Myc	this study
pLG36	pRS306	pSAE2-SAE2-6A-9Myc	this study
pLG37	pRS306	pSAE2-SAE2-14A-9Myc	this study
p2632	pGEX	GST-CDK2-Cak1	Princz et al., 2017 (Ref.15)
p2633	pET21	CycA-6His-ΔN170	Princz et al., 2017 (Ref.15)
pKR555	pFA6-nat-NT2	3AID (3x miniAID)	this study
pJH17	pRS306	pSAE2-SAE2-7A-9Myc (6A + S267A)	this study
pJH19	pRS306	pSAE2-SAE2-S267A-9Myc	this study

Supplementary Table 2– Antibodies used in this study for WB and IF experiments

Antibody	Source	Identifier	dilution	Use
Anti-FLAG (HRP)	Sigma	A8592	1:3000	WB
Anti-Myc	Millipore	05-724	1:2000	WB
Anti-Dbf4	Santa Cruz Biotechnology	Sc-5705	1:2000	WB
Anti-AID (miniAID)	MBL/Biozol	M214-3	1:1000	WB
Anti-Sld2	Philip Zegerman lab.	PZ45	1:2000	WB
Anti-Pgk1	Abcam	ab113687	1:5000	WB
Anti-GAPDH	Abcam	ab9485	1:2500	WB
Anti-γH2AX	Merck	05-636	1:1000	WB
Anti-MCM2 (phospho S53)	Abcam	ab109133	1:1000	WB
secondary anti-mouse (HRP)	Jackson Immuno Research	115-035-003	1:3000	WB
secondary anti-rabbit (HRP)	Jackson Immuno Research	111-035-045	1:3000	WB
secondary anti-goat (HRP)	Jackson Immuno Research	705-035-147	1:3000	WB
anti-RPA70	Abcam	ab79398	1:1000	IF
secondary anti-rabbit Alexa Fluor 488	Invitrogen	A-11008	1:500	IF

Supplementary Table 3 – Sample names for the deposited files from phospho-proteomic experiment

Sample Name	Sample
Orbi3279Sample9	WT replicate 1 (phospho-proteome)
Orbi3279Sample10	WT replicate 2 (phospho-proteome)
Orbi3279Sample11	WT replicate 3 (phospho-proteome)
Orbi3279Sample12	WT replicate 4 (phospho-proteome)
Orbi3279Sample13	*bob1-1* replicate 1 (phospho-proteome)
Orbi3279Sample14	*bob1-1* replicate 2 (phospho-proteome)
Orbi3279Sample15	*bob1-1* replicate 3 (phospho-proteome)
Orbi3279Sample16	*bob1-1* replicate 4 (phospho-proteome)
Orbi3279Sample17	*bob1-1 dbf4*Δ replicate 1 (phospho-proteome)
Orbi3279Sample18	*bob1-1 dbf4*Δ replicate 2 (phospho-proteome)
Orbi3279Sample19	*bob1-1 dbf4*Δ replicate 3 (phospho-proteome)
Orbi3279Sample20	*bob1-1 dbf4*Δ replicate 4 (phospho-proteome)
Orbi3256Sample9	WT replicate 1 (proteome)
Orbi3256Sample10	WT replicate 2 (proteome)
Orbi3256Sample11	WT replicate 3 (proteome)
Orbi3256Sample12	WT replicate 4 (proteome)
Orbi3256Sample13	*bob1-1* replicate 1 (proteome)
Orbi3256Sample14	*bob1-1* replicate 2 (proteome)
Orbi3256Sample15	*bob1-1* replicate 3 (proteome)
Orbi3256Sample16	*bob1-1* replicate 4 (proteome)
Orbi3256Sample17	*bob1-1 dbf4*Δ replicate 1 (proteome)
Orbi3256Sample18	*bob1-1 dbf4*Δ replicate 2 (proteome)
Orbi3256Sample19	*bob1-1 dbf4*Δ replicate 3 (proteome)
Orbi3256Sample20	*bob1-1 dbf4*Δ replicate 4 (proteome)
