# Supplementary material for: Dbf4-dependent kinase promotes cell cycle controlled resection of DNA double-strand breaks and repair by homologous recombination
Source: Nat Commun. 2024 Apr 3;15:2890. doi: 10.1038/s41467-024-46951-z (PMC10991553; doi:10.1038/s41467-024-46951-z)
Supplement: Supplementary file 3 — Description of Additional Supplementary Files [file 41467_2024_46951_MOESM3_ESM.pdf]

Description of Additional Supplementary Files

File Name: Supplementary Data 1
Description: Gene ontology (GO) analysis of biological processes (BP) for proteins part of the DDK-cluster of Figure 1d. The list of gene names from Perseus was used as an input and the analysis was performed with https://geneontology.org using default settings as reported in the excel file (for *p* values a Fisher's exact test with FDR correction was selected).

File Name: Supplementary Data 2
Description: List of the yeast strains used in this study.

File Name: Supplementary Data 3
Description: List of the primers used for qPCR-based experiments in this study.
