# Supplementary material for: Dbf4-dependent kinase promotes cell cycle controlled resection of DNA double-strand breaks and repair by homologous recombination
Source: Nat Commun. 2024 Apr 3;15:2890. doi: 10.1038/s41467-024-46951-z (PMC10991553; doi:10.1038/s41467-024-46951-z)
Supplement: Supplementary file 7 — Reporting Summary [file 41467_2024_46951_MOESM7_ESM.pdf]

nature portfolio

Corresponding author(s): Boris Pfander

Last updated by author(s): Jan 14, 2024

Reporting Summary

Nature Portfolio wishes to improve the reproducibility of the work that we publish. This form provides structure for consistency and transparency in reporting. For further information on Nature Portfolio policies, see our Editorial Policies and the Editorial Policy Checklist.

Statistics

For all statistical analyses, confirm that the following items are present in the figure legend, table legend, main text, or Methods section.

n/a	Confirmed	
☐	☒	The exact sample size (*n*) for each experimental group/condition, given as a discrete number and unit of measurement
☐	☒	A statement on whether measurements were taken from distinct samples or whether the same sample was measured repeatedly
☐	☒	The statistical test(s) used AND whether they are one- or two-sided<br>*Only common tests should be described solely by name; describe more complex techniques in the Methods section.*
☒	☐	A description of all covariates tested
☒	☐	A description of any assumptions or corrections, such as tests of normality and adjustment for multiple comparisons
☐	☒	A full description of the statistical parameters including central tendency (e.g. means) or other basic estimates (e.g. regression coefficient) AND variation (e.g. standard deviation) or associated estimates of uncertainty (e.g. confidence intervals)
☐	☒	For null hypothesis testing, the test statistic (e.g. *F*, *t*, *r*) with confidence intervals, effect sizes, degrees of freedom and *P* value noted<br>*Give P values as exact values whenever suitable.*
☒	☐	For Bayesian analysis, information on the choice of priors and Markov chain Monte Carlo settings
☒	☐	For hierarchical and complex designs, identification of the appropriate level for tests and full reporting of outcomes
☒	☐	Estimates of effect sizes (e.g. Cohen's *d*, Pearson's *r*), indicating how they were calculated

Our web collection on statistics for biologists contains articles on many of the points above.

Software and code

Policy information about availability of computer code

Data collection	Fujifilm Image Reader LAS-3000 (v2.2)<br>MACSQuantify (v2.11)<br>Light Cycler 480 (v1.5.0.39)<br>Typhoon 9500 phosphorimager<br>Invitrogen EVOS FL Auto2 Imaging System
Data analysis	FlowJo (v10.5.3)<br>FALCOR (lianglab.brocku.ca/FALCOR/)<br>t-test calculator ((https://www.graphpad.com/quickcalcs/ttest1.cfm)<br>Graphpad Prism 8<br>Cell Profiler (Version 4.2.5)<br>R version 4.3.1<br>R version 4.2.2<br>R version 3.6.2<br>Bowtie2 (v2.4.2)<br>SAMtools (v1.9)<br>bedtools (v2.30.0)<br>MaxQuant computational platform

Perseus (v1.6.15.0)
DNASTAR Lasergene (v15 and v17)
Light Cycler 480 software release 1.5.0 SP4 (v1.5.0.39)
WebLogo (v2.8.2) (https://weblogo.berkeley.edu/logo.cgi)
Panther (geneontology.org)
Fiji-ImageJ (v.2.14.0/1.54f)

For manuscripts utilizing custom algorithms or software that are central to the research but not yet described in published literature, software must be made available to editors and reviewers. We strongly encourage code deposition in a community repository (e.g. GitHub). See the Nature Portfolio guidelines for submitting code & software for further information.

Data

Policy information about availability of data

All manuscripts must include a data availability statement. This statement should provide the following information, where applicable:

- Accession codes, unique identifiers, or web links for publicly available datasets
- A description of any restrictions on data availability
- For clinical datasets or third party data, please ensure that the statement adheres to our policy

ChIP-seq data is available at NCBI GEO under accession number: GSE233549. Mass spectrometry data is available at EBI-PRIDE under accession number: PXD042607. For NGS experiments, the S. cerevisiae LYZE00000000.1 genome assembly (PMID: 28584079 (https://pubmed.ncbi.nlm.nih.gov/28584079/)) was used for mapping of sequencing reads after removing the sequences of the HML and HMR loci, which are deleted in all the strains used for NGS experiments. For Mass spectrometry experiments , the peak list was searched against the reviewed Uniprot yeast proteome database (proteome ID: UP000002311 (https://www.uniprot.org/proteomes/UP000002311); downloaded in 2019). see methods section for details.

Research involving human participants, their data, or biological material

Policy information about studies with human participants or human data. See also policy information about sex, gender (identity/presentation), and sexual orientation and race, ethnicity and racism.

Reporting on sex and gender	N/A
Reporting on race, ethnicity, or other socially relevant groupings	N/A
Population characteristics	N/A
Recruitment	N/A
Ethics oversight	N/A

Note that full information on the approval of the study protocol must also be provided in the manuscript.

Field-specific reporting

Please select the one below that is the best fit for your research. If you are not sure, read the appropriate sections before making your selection.

☒ Life sciences ☐ Behavioural & social sciences ☐ Ecological, evolutionary & environmental sciences

For a reference copy of the document with all sections, see nature.com/documents/nr-reporting-summary-flat.pdf

Life sciences study design

All studies must disclose on these points even when the disclosure is negative.

Sample size	For experiment with yeast cells, all data are from ensemble experiments from at least 10.000.000 budding yeast cells. No sample size calculation was performed. Previous publications showed that that such cell numbers are large enough to reproducibly reveal attributes of yeast cells ensembles; for flow cytometry experiments at least 10.000 cells were analyzed (see for example: Lademann et al., 2017 (PMID: 28514650); Bantele et al., 2017 (PMID: 28063255); Peritore et al., 2021 (PMID: 33651987); Reusswig et al., 2022 (PMID: 36400763)). For experiments with U2OS cells, no sample size calculation was performed. The number of seeded cells was tested to allow reproducible detection of proteins via WB. Moreover, it was tested to allow (after fixation and processing) the measurement of DNA content of around 10.000 cells via FACS, a number generally accepted to allow visualization of smooth peaks (see for example Crowley et al., 2016 (PMID: 27698234)). Within the different samples we could measure between 7.500 and 25.000 cells. For IF experiments, no sample size calculation was performed before staining/image collection. For different samples at least 60 mitotic nuclei were analyzed providing sufficient data points for statistical analysis via two-sided Mann-Whitney test.
Data exclusions	Pre-established criteria were used to select NGS and Phosphoproteomic data for further analysis:<br>NGS: Reads mapping to repetitive regions and ambiguously mapping reads were excluded from analysis.

Phosphoproteomics: Whole proteome were analysed side-by-side, phospho-peptides were excluded when derived from proteins that showed significant changes after ANOVA test comparing whole proteome in different strains.
See Methods section for further details.

Replication
All experiments were performed in at least two biological replicates, except for experiment in Supplementary Figure 2l which was performed in two technical replicates. All replications showed reproducible results.

Randomization
Randomization was not relevant to this study since samples did not need to be allocated to specific groups.

Blinding
Blinding was not relevant because there was no group allocation during data collection or analysis.

Reporting for specific materials, systems and methods

We require information from authors about some types of materials, experimental systems and methods used in many studies. Here, indicate whether each material, system or method listed is relevant to your study. If you are not sure if a list item applies to your research, read the appropriate section before selecting a response.

Materials & experimental systems

n/a	Involved in the study
☐	☒ Antibodies
☐	☒ Eukaryotic cell lines
☒	☐ Palaeontology and archaeology
☒	☐ Animals and other organisms
☒	☐ Clinical data
☒	☐ Dual use research of concern
☒	☐ Plants

Methods

n/a	Involved in the study
☐	☒ ChIP-seq
☐	☒ Flow cytometry
☒	☐ MRI-based neuroimaging

Antibodies

Antibodies used
Anti-FLAG (HRP), Sigma (A8592, clone M2); Anti-Myc, Millipore (05-724, clone 4A6); Anti-Dbf4, Santa Cruz Biotechnology (Sc-5705); Anti-AID (miniAID), MBL/Biozol (M214-3, clone 1E4); Anti-Sld2, Philip Zegerman lab (PZ45); Anti-Pgk1, Abcam (ab113687, clone 22C5D8); Anti-GAPDH, Abcam (ab9485); Anti-MCM2 (phospho S53), Abcam (ab109133, clone EP4120); Anti-gammaH2AX, Merck (05-636, clone JBW301); Anti-RPA70, Abcam (ab79398, cloneEPR3472); anti-RFA antibody, Agrisera (AS07 214); secondary anti-mouse (HRP), Jackson Immuno Research (115-035-003); secondary anti-rabbit (HRP), Jackson Immuno Research (111-035-045); secondary anti-goat (HRP), Jackson Immuno Research (705-035-147); secondary anti-rabbit Alexa Fluor 488, Imvitrogen (A-11008).

Validation
Anti-FLAG-HRP (clone M2, Sigma, A8592):
Monoclonal ANTI-FLAG® M2-Peroxidase (HRP) antibody produced in mouse. The antibody was validated by the company for binding to FLAG fusion proteins and recognizing the FLAG epitope at any position in the fusion protein. Reported by the company to be useful for identification of FLAG fusion proteins (also) by western blot. Used in this study for western blot.

Anti-Myc (clone 4A6, Millipore, 05-724)
Anti-Myc Tag, clone 4A6, is a mouse monoclonal antibody that is validated by the company for the detection of Myc Tag in ChIP, IC, IF, IP, and western blot. Used in this study for western blot.

Anti-Dbf4 (Sc-5705, Santa Cruz Biotechnology)
Goat polyclonal antibody raised against a peptide mapping close to the N-terminus of Dbf4 of S. cerevisiae origin. Recommended by the company for detection of Dbf4 from S. cerevisiae (also) by western blot. Previously used for detection of S. cerevisiae Dbf4 in different publications, such as Reusswig et al., 2016 (PMID: 27705801); Seoane and Morgan., 2017 (PMID: 28918948); Princz et al., 2017 (PMID: 28096179); Lao et al., 2018 (PMID: 30377154). Used in this study for western blot.

Anti-AID (mini-AID) (clone 1E4, MBL/Biozol, M214-3)
Mouse monoclonal antibody recognizing a 17 amino acids sequence of the Auxin inducible Degron internal region, allowing detection of mini-AID tagged proteins. Validated from the company to be used (also) for western blot. Used in this study for western blot.

Anti-Sld2 (from Philip Zegerman laboratory, PZ45)
Rabbit polyclonal antibody which was validated in previous publications (such as Reusswig et al., 2016 (PMID: 27705801)) for detection of Sld2 from S. cerevisiae via western blot. Used in this study for western blot.

Anti-Pgk1 (clone 22C5D8, Abcam, ab113687)
Mouse Monoclonal antibody recognizing Pgk1 from S. cerevisiae. Tested by the company to be used in western blot in S. cerevisiae samples. Used in different publications such as Liu et al., 2023 (PMID: 37349354); Romanauska and Köhler., 2023 (PMID: 37591950). Used in this study for western blot.

Anti-GAPDH (Abcam, ab9485)
Rabbit Polyclonal GAPDH antibody. Validated by the company to be used for detection of human GAPDH (also) with western blot and used in different publications such as Liu et al., 2023 (PMID: 38040699) Nat Comms and Große-Segerath et al., 2024 (PMID:

38316785). Used in this study for western blot.

Anti-gammaH2AX (clone JBW301, Merck, 05-636)
Mouse monoclonal antibody which recognizes human histone H2A.X phosphorylated at serine 139.
Immunogen: peptide (C-KATQA[pS]QEY) corresponding to amino acids 134-142 of human histone H2A.X. Validated by the company to be suitable (also) for western blot. Used in previous publications such as Leung et al., 2017 (PMID: 28242625); Sanchez-Bailon et al., 2021 (PMID: 34728620). Used in this study for western blot.

Anti-MCM2 (phospho S53) (clone EP4120, Abcam, ab109133)
Rabbit recombinant monoclonal MCM2 phospho S53 antibody. Tested by the company for detection of human MCM2 phosphorylated on serine 53 (also) by western blot. Used in different publications, such as Wang et al, 2019 (PMID: 31578521); Wienert et al., 2020 (PMID: 32355159). Used in this study for western blot.

Anti-RPA70 (clone EPR3472, Abcam, ab79398)
Rabbit Recombinant Monoclonal RPA70 antibody. Validated by the company (also) for immunofluorescence and tested in human samples. Used in previous publications, such as Lezaja et al., 2021 (PMID: 34158486). Used in this study for immunofluorescence.

Anti-RFA (Agrisera, AS07 214)
Rabbit polyclonal anti-RFA antibody. Confirmed by the company for reactivity against S. cerevsiaie RFA. Immunogen: RPA from S. cerevisiae consisting of three subunits RFA1 (70 kDa), RFA2 (30 kDa) and RFA3 (14 kDa). Confirmed by the company to be used (also) for chromatin immunoprecipitation. Used in different publications such as: Holstein et al., 2014 (PMID: 24835988); Bantele et al., 2017 (PMID: 28063255); Peritore et al., 2021 (PMID: 33651987). Used in this study for chromatin immunoprecipitation.

Eukaryotic cell lines

Policy information about cell lines and Sex and Gender in Research

Cell line source(s)	Yeast strains from the Pfander laboratory collection, listed in Supplementary Data 2. Yest strains YLG510, 512, 515 are derivatives of yeast strains received from M.A. Resnick laboratory (Lobachev et al., 2002). Strains LSY5474-8B, LSY5474-18D, LSY5575-106C, LSY5575-82A, LSY5602-1D, LSY5575-91D, LSY5949-4A, LSY5949-7C were constructed in the laboratory of L. Symington.<br>Human U2OS osteosarcoma cell line was a kind gift from the laboratory of Ralf Jungmann.
Authentication	Gene deletions and single plasmid integrations in yeast strains were confirmed by PCR. Gene tagging was confirmed by PCR and western blot. Introduction of point mutations in yeast strains was confirmed by sequencing. U2OS cell line was not further authenticated.
Mycoplasma contamination	Human U2OS osteosarcoma cells were used only within less than 10 passages and thus not tested for mycoplasma contamination.
Commonly misidentified lines<br>(See ICLAC register)	No commonly misidentified cell lines were used

Plants

Seed stocks	*Report on the source of all seed stocks or other plant material used. If applicable, state the seed stock centre and catalogue number. If plant specimens were collected from the field, describe the collection location, date and sampling procedures.*
Novel plant genotypes	*Describe the methods by which all novel plant genotypes were produced. This includes those generated by transgenic approaches, gene editing, chemical/radiation-based mutagenesis and hybridization. For transgenic lines, describe the transformation method, the number of independent lines analyzed and the generation upon which experiments were performed. For gene-edited lines, describe the editor used, the endogenous sequence targeted for editing, the targeting guide RNA sequence (if applicable) and how the editor was applied.*
Authentication	*Describe any authentication procedures for each seed stock used or novel genotype generated. Describe any experiments used to assess the effect of a mutation and, where applicable, how potential secondary effects (e.g. second site T-DNA insertions, mosiacism, off-target gene editing) were examined.*

ChIP-seq

Data deposition

☒ Confirm that both raw and final processed data have been deposited in a public database such as GEO.

☐ Confirm that you have deposited or provided access to graph files (e.g. BED files) for the called peaks.

Data access links<br>*May remain private before publication.*	https://www.ncbi.nlm.nih.gov/geo/query/acc.cgi?acc=GSE233549
Files in database submission	GSE233549_RAW.tar contains files for 96 samples, 2 coverage bedgraph-files (forward and reverse strand) and 2 raw data files (paired-end reads)

Genome browser session (e.g. UCSC)	see link above, data are available on GEO with the token: ahireiwqdponfgv

Methodology

Replicates	n=4 for experiments shown in figure 2 and S2; n=2 for experiments shown in figure 5 and S5.
Sequencing depth	+/- 10 million reads per sample, paired-end, 75 bp on Illumina NextSeq 500 or 60 bp on Illumina NovaSeq 6000
Antibodies	anti-RFA antibody (Agrisera, AS07214)
Peak calling parameters	unspecific RPA peaks for normalization were selected using the function “slice” with the lower value set to 6x the median coverage from the R package IRanges (v2.32.0). Only peaks with a width between 100 and 500 bp and overlapping between different samples and replicates of the same type of experiment (arrest in G1 phase or in M phase of the cell cycle) were retained.
Data quality	Quality of raw data was assessed using fastQC (v 0.11.7)
Software	R version 4.2.2<br>IRanges (v2.32.0)<br>Bowtie2 (v2.4.2)<br>SAMtools (v1.9)<br>bedtools (v2.30.0)

Flow Cytometry

Plots

Confirm that:

- [x] The axis labels state the marker and fluorochrome used (e.g. CD4-FITC).
- [ ] The axis scales are clearly visible. Include numbers along axes only for bottom left plot of group (a 'group' is an analysis of identical markers).
- [ ] All plots are contour plots with outliers or pseudocolor plots.
- [ ] A numerical value for number of cells or percentage (with statistics) is provided.

Methodology

Sample preparation	Briefly, yeast cells were fixed and permeabilized in 70% ethanol + 50 mM Tris-HCl pH 8.0, digested with RNase A and proteinase K and stained with 0.5 µM SYTOX green (Invitrogen, S7020).<br>For U2OS cells, briefly, cells were fixed with Methanol, then washed with PBS 1X; 0.01% Triton X-100 and incubated with propidium iodide buffer (PBS 1X; 0.01% Triton X-100; 10 µg/ml RNase A; 25 µg/ml propidium iodide(P3566, Invitrogen)) before measurement.<br>See Methods section for details.
Instrument	MACSquant Analyzer 10 (Milteny Biotec)
Software	FlowJo (v10.5.3)
Cell population abundance	no gating was performed.
Gating strategy	no gating was performed.

- [ ] Tick this box to confirm that a figure exemplifying the gating strategy is provided in the Supplementary Information.
